# Evaluating anti-viral effect of Tylvalosin tartrate on porcine reproductive and respiratory syndrome virus and analyzing the related gene regulation by transcriptomics

**DOI:** 10.1186/s12985-023-02043-w

**Published:** 2023-04-26

**Authors:** Xingzhen Tang, Cong Wang, Weifeng Sun, Weixin Wu, Shaohui Sun, Jin Wan, Guangshan Zhu, Nini Ma, Xiaoping Ma, Ruihua Xu, Qiushi Yang, Yindi Dai, Lei Zhou

**Affiliations:** 1 National Key Laboratory of Veterinary Public Health Security, College of Veterinary Medicine, China Agricultural University, No. 2 Yuanmingyuan West Road, Haidian District, Beijing, 100193 People’s Republic of China; 2 Key Laboratory of Animal Epidemiology of Ministry of Agriculture and Rural Affairs, College of Veterinary Medicine, China Agricultural University, Beijing, 100193 People’s Republic of China; 3 China Animal Husbandry Industry Co., Ltd, Beijing, 100070 People’s Republic of China; 4 China Animal Nanjing Veterinary Drugs Co., Ltd, Nanjing, 210012 People’s Republic of China

**Keywords:** Porcine reproductive and respiratory syndrome virus (PRRSV), Tylvalosin, Anti-virus, Transcriptome analysis

## Abstract

**Background:**

Porcine reproductive and respiratory syndrome virus (PRRSV) is an economically important pathogen, characterized by its genetic and antigenic variation. The PRRSV vaccine is widely used, however, the unsatisfied heterologic protection and the risk of reverse virulence raise the requirement to find some new anti-PRRSV strategies for disease control. Tylvalosin tartrate is used to inhibit PRRSV in the field non-specifically, however, the mechanism is still less known.

**Methods:**

The antiviral effects of Tylvalosin tartrates from three producers were evaluated in a cell inoculation model. Their safety and efficacy concentrations, and effecting stage during PRRSV infection were analyzed. And, the Tylvalosin tartrates regulated genes and pathways which are potentially related to the anti-viral effect were further explored by using transcriptomics analysis. Last, the transcription level of six anti-virus-related DEGs was selected to confirm by qPCR, and the expression level of HMOX1, a reported anti-PRRSV gene, was proved by western blot.

**Results:**

The safety concentrations of Tylvalosin tartrates from three different producers were 40 µg/mL (Tyl A, Tyl B, and Tyl C) in MARC-145 cells and 20 µg/mL (Tyl A) or 40 µg/mL (Tyl B and Tyl C) in primary pulmonary alveolar macrophages (PAMs) respectively. Tylvalosin tartrate can inhibit PRRSV proliferation in a dose-dependent manner, causing more than 90% proliferation reduction at 40 µg/mL. But it shows no virucidal effect, and only achieves the antiviral effect via long-term action on the cells during the PRRSV proliferation. Furthermore, GO terms and KEGG pathway analysis was carried out based on the RNA sequencing and transcriptomic data. It was found that the Tylvalosin tartrates can regulate the signal transduction, proteolysis, and oxidation-reduction process, as well as some pathways such as protein digestion and absorption, PI3K-Akt signaling, FoxO signaling, and Ferroptosis pathways, which might relate to PRRSV proliferation or host innate immune response, but further studies still need to confirm it. Among them, six antivirus-related genes HMOX1, ATF3, FTH1, FTL, NR4A1, and CDKN1A were identified to be regulated by Tylvalosin tartrate, and the increased expression level of HMOX1 was further confirmed by western blot.

**Conclusions:**

Tylvalosin tartrate can inhibit PRRSV proliferation in vitro in a dose-dependent manner. The identified DEGs and pathways in transcriptomic data will provide valuable clues for further exploring the host cell restriction factors or anti-PRRSV target.

## Introduction

Porcine reproductive and respiratory syndrome (PRRS) is an economically important swine disease that seriously hinders porcine health and wellbeing, which has been an epidemic for more than 30 years since it was first reported in the United States in 1987 [1, 2]. PRRS can cause reproductive disorders in pregnant sows, showing as premature delivery, abortion, stillbirth, mummified fetus, weak piglets, as well as respiratory diseases in all age pigs [3]. Furthermore, due to the properties of immunosuppression, PRRS can lead to increased secondary infections in pigs, such as *Mycoplasma suis*, *Streptococcus suis*, *actinobacillus pleuropneumoniae*, porcine respiratory coronavirus, and porcine circovirus type 2 (PCV2) [4–8]. In 2006, atypical PRRS, caused by highly pathogenic PRRSV (HP-PRRSV), outbroke in China and some other Asian countries, which results in more serious clinical symptoms than early strains, including high fever (up to 42 °C), high morbidity and mortality in pigs [9, 10]. Recently, in the United States, the Lineage 1 C variant strains with the 1-4-4 pattern of restriction fragment length polymorphism (RFLP) in the open reading frame 5 (ORF5) have been found to show a similar fatal virulence of HP-PRRSV [11]. Both events have deepened the understanding of PRRSV pathogenicity. Meanwhile, PRRSV is a single-stranded positive RNA virus with low replication fidelity [12], and it is also characterized by its genetic and antigenic variation. Serial previous studies have reported that the commercial PRRSV vaccine can only provide limited heterologic protection against the current field strains, meanwhile, the mutation and recombination greatly increased the diversity of field strains, which makes the prevention and control more complicated [13–15]. Besides, the modified live virus (MLV) vaccine has the risk of reverse to virulence and recombination with field strains [13, 16, 17]. Therefore, the development of new anti-PRRSV strategies is of great significance for disease prevention and control.

Macrolides are a class of antibiotics that consist of a large macrocyclic lactone ring, which can bind to bacterial ribosomal 50s subunits and inhibits bacterial protein synthesis [18, 19]. Tylvalosin is the third generation of a macrolide antibiotic, which is a derivative of Tylosin and modified by 3-acetyl-4-isovaleryl, with the chemical formula C_53_H_87_NO_19_ and a molecular weight of 1042.3 [19]. Tylvalosin is widely used to treat respiratory and intestinal diseases such as mycoplasma, spirochete, and intracellular Lawson infection in pigs and poultry [20, 21].

In a previous study, it has been reported that Tylvalosin tartrate treatment could significantly reduce the incidence of pneumonia in pigs inoculated with *Mycoplasmapneumoniae* and *Pasteurella multocida* [21]. Besides, it is also regarded that Tylvalosin tartrate has the property of non-specifically inhibiting PRRSV proliferation both in vitro and in vivo, and nowadays it has been commonly used on pig farms for both reducing the impact of secondary bacterial infection and inhibiting PRRSV replication in pigs [22, 23]. However, the inhibition mechanism is still less known. To verify its anti-PRRSV effect and explore the possible mechanism, a cell inoculation model was set to evaluate the commercial products of Tylvalosin tartrate from three different producers. The antiviral effects at different concentrations and viral infection stages were analyzed, as well, the potential antiviral mechanisms were further explored by using transcriptomic analysis in this study.

## Materials and methods

### Cells and viruses

MARC-145 cells were grown in Dulbecco’s modified Eagle’s medium (DMEM) (ThermoFisher, CA, USA) with 10% fetal bovine serum (FBS) and penicillin and streptomycin at 37 °C in a humidified atmosphere of 5% CO_2_. As the previous description, the primary pulmonary alveolar macrophages (PAMs) were prepared from 4-week-old specific-pathogen-free (SPF) landrace pigs and cultured in RPMI-1640 ThermoFisher, CA, USA medium with 10% FBS and penicillin and streptomycin at 37 °C in a humidified atmosphere of 5% CO_2_ [24]. The protocol for primary PAMs preparation was approved by the Laboratory Animal Ethical Committee of China Agricultural University, with approval No. AW81801202-2-1. The HP-PRRSV strain JXwn06 (GenBank accession number EF641008) and NADC30-like strain CHsx1401 (GenBank accession number KP861625) at the 8th passage were used in this study. A recombinant PRRSV strain RvJX-Nsp2_325_-HiBiT, carrying a complementary luciferase subunit from NanoLuc luciferase, was constructed in our previous study, which can be easily used to evaluate the efficacy of antiviral reagents by detecting the reduction of luciferase activity, showing a consistent trend with infectious titers [25].

### Cell viability assay

Tylvalosin tartrate soluble powder (Tyl A, Tyl B, and Tyl C) from three different producers were suspended in sterile water at a concentration of 2 mg/mL respectively and then filtered with a 0.22 μm filter. Then the cytotoxicity of Tylvalosin tartrate on MARC-145 cells and primary PAMs was assessed by cell proliferation assay with Cell Counting Kit-8 (CCK-8). Briefly, MARC-145 cells or PAMs were treated with each reagent at a range of concentrations (1, 10, 20, 40, 50, and 100 µg/mL) and incubated at 37 °C for 36 h. The DMEM cell culture medium was set as the negative control. Cell viability was tested by using the Cell Proliferation and Cytotoxic Assay Kit (Solarbio, Beijing, China) following the manufacturer’s instructions.

### Evaluating the inhibiting effect of Tylvalosin tartrate on PRRSV

MARC-145 cells were seeded in a 96-well plate (1 × 10^4^ cells/well) and grown to confluence in DMEM with 10% FBS. The filtered Tylvalosin tartrate stocks were diluted with DMEM containing 2% FBS to prepare the working solution with the concentration of 1, 10, 20, and 40 µg/mL, and then treated the cells for 1 h before viral inoculation. Meanwhile, the virus stock of RvJX-Nsp2_325_-HiBiT was also diluted with the Tylvalosin tartrate solution to the same concentration above. The Tylvalosin tartrate pretreated cells were inoculated with diluted RvJX-Nsp2_325_-HiBiT with the multiplicity of infection (MOI) at 0.1. After 36 h of incubation at 37 °C in a humidified atmosphere of 5% CO_2_, the cells were harvested by frozen at − 80 °C and thawed, then the luciferase activity of the supernatant was measured by using a Nano-Glo^®^ HiBiT lytic detection system (Promega, Madison, WI, USA) according to the manufacturer’s instructions, and the generated luminescence was detected by the luminometer after 10 min reaction [25].

To further confirm the inhibition effect, MARC-145 cells and the PRRSV target cell PAMs were both inoculated with HP-PRRSV strain JXwn06 or currently predominant NADC30-like virus CHsx1401, respectively, with the treatment of Tylvalosin tartrate (40 µg/mL). After 36 h inoculation, the cells were fixed with cold ethanol and examined by indirect immunofluorescence assay (IFA) using the PRRSV anti-N monoclonal antibody (McAb) SDOW17 [26].

### Detecting the inhibition stages of Tylvalosin tartrate on PRRSV

**Fig. 1:**
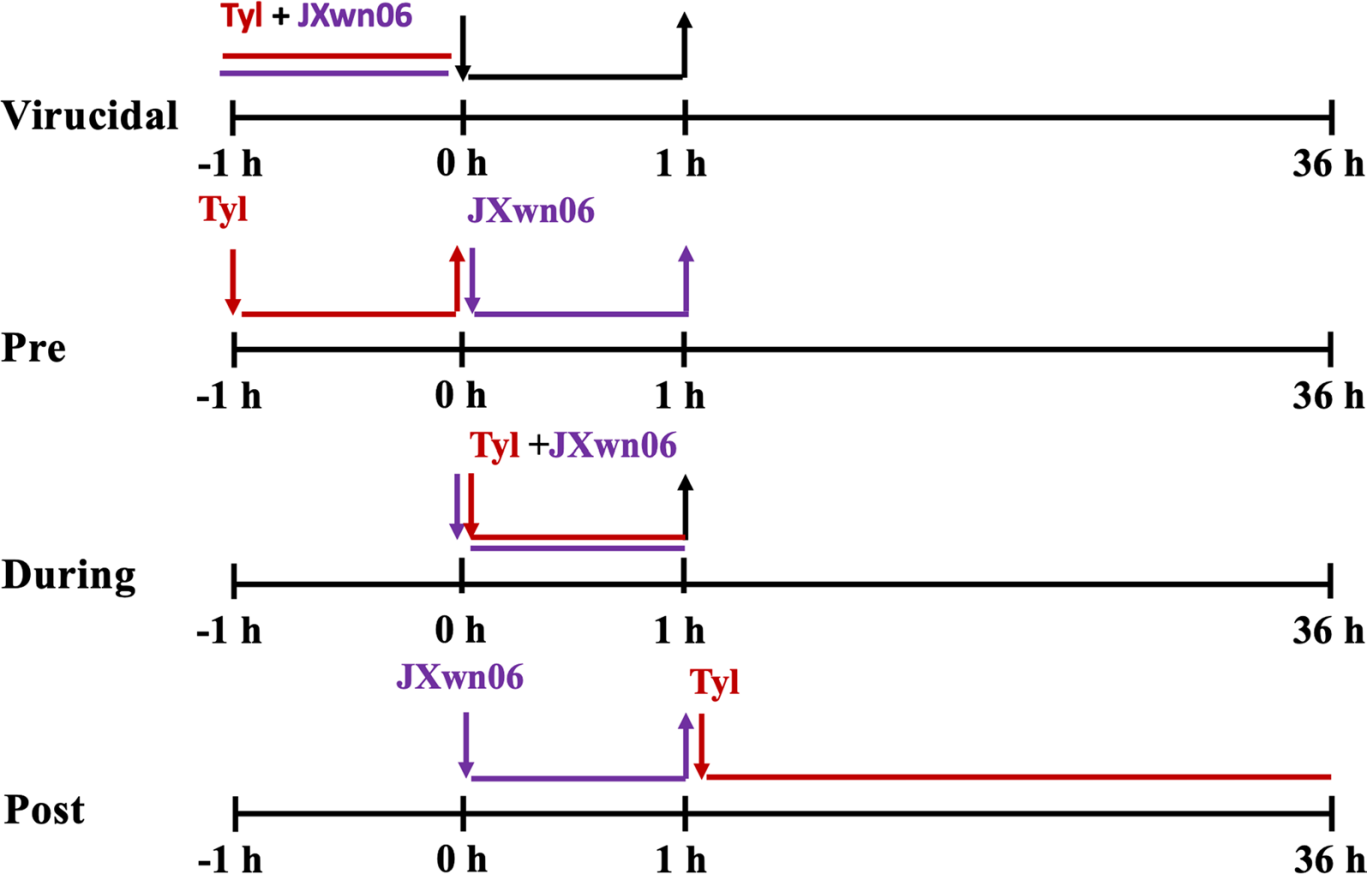
The schematic diagram for the process of Tylvalosin tartrate treatment at different PRRSV infection stages

MARC-145 cells were seeded in a 48-well plate and grow to confluence in DMEM with 10% FBS. Four groups with different Tylvalosin tartrate treating stages were set as follows (Fig. 1). To test the virucidal effect of Tylvalosin tartrate, the JXwn06 virus was pretreated with Tylvalosin tartrate (Tyl A, 40 µg/mL) for 1 h at 37 °C, then added to MARC-145, after another 1 h incubation at 37 °C, the cells were washed with PBS for 3 times and maintained in 2% FBS DMEM without Tylvalosin tartrate. Parallelly, Pre-, During- and Post-inoculation treating groups were also set. After 36 h incubation at 37 °C in a humidified atmosphere of 5% CO_2_, the cells were harvested by frozen at − 80 °C and thawed. The titers of JXwn06 in each group were determined by endpoint assay based on IFA and shown as TCID_50_/mL calculated by the Reed-Müench method.

### Preparing the cells for transcriptomic analysis

To investigate the differentially expressed genes and pathways related to the treatment of Tylvalosin tartrate, cells from four different groups were submitted for transcriptomic analysis, which included groups of non-treated MARC-145 cells, Tylvalosin tartrate treatment, PRRSV infection, and PRRSV infection with Tylvalosin tartrate treatment. Triple independent repeats were set in each group. At 24 h post-inoculation, the cells were harvested by adding TRIzol reagents and stored at − 80 °C.

### Transcriptomic sequencing

The total RNA of MARC-145 cells from different groups was extracted by using TRIzol reagent (ThermoFisher, CA, USA) following the manufacturer’s procedure. RNA library construction and sequencing were then performed by LC Bio-Technology Co., Ltd (Zhejiang, China). Briefly, the purity and quantity of the total RNA were analyzed by using Bioanalyzer 2100 and RNA 6000 Nano LabChip Kit (Agilent, CA, USA), and the high-quality RNA samples with RIN number > 7.0 were selected to construct a sequencing library. The mRNA was enriched from the total RNA (5 µg) by using Dynabeads Oligo (dT) (ThermoFisher, CA, USA) with two rounds of purification. The purified mRNA was then fragmented into small pieces using divalent cations under elevated temperatures. Following this step, the cleaved RNA fragments were reverse-transcribed into cDNA by SuperScript™ II Reverse Transcriptase (ThermoFisher, CA, USA), which were further used to synthesize U-labeled second-stranded DNAs with *E. coli* DNA polymerase I (NEB, Beijing, China), RNase H (NEB, Beijing, China) and dUTP Solution (ThermoFisher, CA, USA). Then one A-base nucleotide was added to the blunt ends of each strand for ligation to the indexed adapters, which contain a T-base overhang for ligating. The fragments with dual-index adapters were selected based on the size of AMPure XP beads. After the treatment of heat-labile UDG enzyme (NEB, M0280), the U-labeled second-stranded DNAs were amplified by PCR with the following conditions: initial denaturation at 95 °C for 3 min; 8 cycles of denaturation at 98 °C for 15 s, annealing at 60 °C for 15s, and extension at 72 °C for 30 s; and then final extension at 72 °C for 5 min. The average insert size for the final cDNA libraries was 300 bp ± 50 bp. Finally, 2 × 150 bp paired-end sequencing (PE150) was performed on an Illumina Novaseq™ 6000 (LC Bio-Technology Co., Ltd. Zhejiang, China) according to the standard protocol, and the raw sequencing data were collected and preserved into FASTQ format. Raw data were further filtered to obtain clean reads by Cutadapt to remove the adaptors, reads with more than 5% of unknown base, and those with low quality. Then the acquired reads were aligned to the *Macaca mulatta* reference genome by using the HISAT2 (https://daehwankimlab.github.io/hisat2/, version: hisat2-2.0.4) package, and the mapped reads of each sample were assembled by using StringTie (http://ccb.jhu.edu/software/stringtie/, version: stringtie-1.3.4d). Subsequently, all transcriptomes from all samples were merged to reconstruct a comprehensive transcriptome using gffcompare software (http://ccb.jhu.edu/software/stringtie/gffcompare.shtml, version: gffcompare-0.9.8). After the final transcriptome was generated, StringTie and ballgown (http://www.bioconductor.org/packages/release/bioc/html/ballgown.html) were used to estimate the expression levels of all transcripts and perform expression abundance for mRNAs by calculating the FPKM (fragment per kilobase of transcript per million mapped reads) value. The differentially expressed mRNAs and genes were selected with log2 (fold change) > 1 or log2 (fold change) <-1 and with statistical significance (*p* value < 0.05) by the R package.

### Venn, GO and KEGG pathway enrichment analysis

Genes differential expression analysis was performed by using DESeq2 software among four different groups (and by edgeR between every two groups). The gene with the parameter of false discovery rate (FDR) below 0.05 and absolute fold change ≥ 2 was considered as a differentially expressed one. Differentially expressed genes (DEGs) were then subjected to enrichment analysis of GO functions and KEGG pathways. Venn analysis, GO enrichment, and KEGG enrichment analysis were performed as described by LC Bio-Technology Co., Ltd. (https://www.lc-bio.cn/).

### Quantitative reverse transcription PCR (RT-qPCR)

MARC-145 cells from four Tylvalosin tartrates treated and/ or PRRSV infection groups mentioned above were collected 24 h post-inoculation, and the total RNA was extracted by using TRIzol following the manufacturer’s instructions. The reverse transcription was carried out by using FastKing RT Kit (with gDNase)(Tiangen, Beijing China), followed by real-time PCR on the Bio-Rad CFX96 thermal cycler (Bio-Rad, CA, USA) with primers listed in Table 1 and AceQ qPCR SYBR Green Master Mix (Vazyme, Nanjing, China ). The thermal protocol is as below: 95 °C for 2 min; followed by 40 cycles of 95 °C for 10 s, and 60 °C for 50 s. The data was collected at the step of 60 °C annealing/elongation. Relative quantification of test genes was calculated by using the 2^−ΔΔCt^ method with GAPDH as a housekeeping gene. Three independent repeats were carried out.

**Table 1:** List of primers used in this study

Names*	Primer sequence (5’-3’)
GAPDH -F	TGATGACATCAAGAAGGTGGTGAAG
GAPDH -R	TCCTTGGAGGCCATGTGGGCCAT
HMOX1-F	CCCTACACACTGGCAATG
HMOX1-R	AGCAATCTTCTTGAGCATCT
ATF3-F	CTAAGCAGCCGTGGTATG
ATF3-R	GCCTTGTTGGTTCTCTG
FTH1-F	ACTACCACCAGGACTCAG
FTH1-R	AAGTTCTTCAAAGCCACATC
FTL -F	CTGTGACTTCCTGGAGACT
FTL -R	GTCGTGCTTGAGAGTGAG
NR4A1-F	CTGCCAATCTCCTCACTTC
NR4A1-R	AGGTCGTAGAACTGCTGTA
CDKN1A -F	CAGACCAGCATGACAGATT
CDKN1A -R	ACTAAGGCAGAAGATGTAGAG

*F represents forward PCR primer; R represents reverse PCR primer

### Western blot

MARC-145 cells from four Tylvalosin tartrates treated and/ or PRRSV infection groups mentioned above were collected 24 h post-inoculation. RIPA lysis buffer (Beyotime, Shanghai, China) supplemented with 1 mM PMSF (Beyotime, Shanghai, China) was used to extract total proteins from treated MARC-145 cells on ice. Protein concentrations were measured with an enhanced BCA protein assay kit (Beyotime, Shanghai, China). Then, 20 ug proteins per sample were mixed with 5 × loading buffer and boiled at 100 °C for 10 min, and they were separated by SDS-PAGE. After transferring the proteins onto polyvinylidene difluoride membranes (Millipore, Darmstadt, Germany), the membrane was blocked in PBS with 5% skimmed milk at room temperature for 3 h. After that, the membrane was incubated at 4 °C overnight with primary antibodies and HRP-conjugated anti-mouse or anti-rabbit secondary antibodies at 4 °C for 3 h. The protein bands were detected by the ECL western blotting system (Thermo Fisher, Waltham, USA). The following primary antibodies were used: anti-HMOX1 (Abmart, Shanghai, China), anti-β-actin (CUSABIO, Hubei, China), and the mAb against PRRSV N protein was kindly provided by professor Ping Jiang (Nanjing Agricultural University, Nanjing, China).

### Statistical analysis

The data of cell viability, viral titers, relative luciferase activity, and FPKM in the transcriptome were displayed as means ± standard deviations (SD). The GraphPad Prism software (version 9.0) was used to determine the significance of the variability among different groups by a two-way ANOVA test of variance. A *p* value < 0.05 was considered to be statistically significant.

## Results

### Safety of Tylvalosin tartrate on MARC-145 cells and primary PAMs

**Fig. 2:**
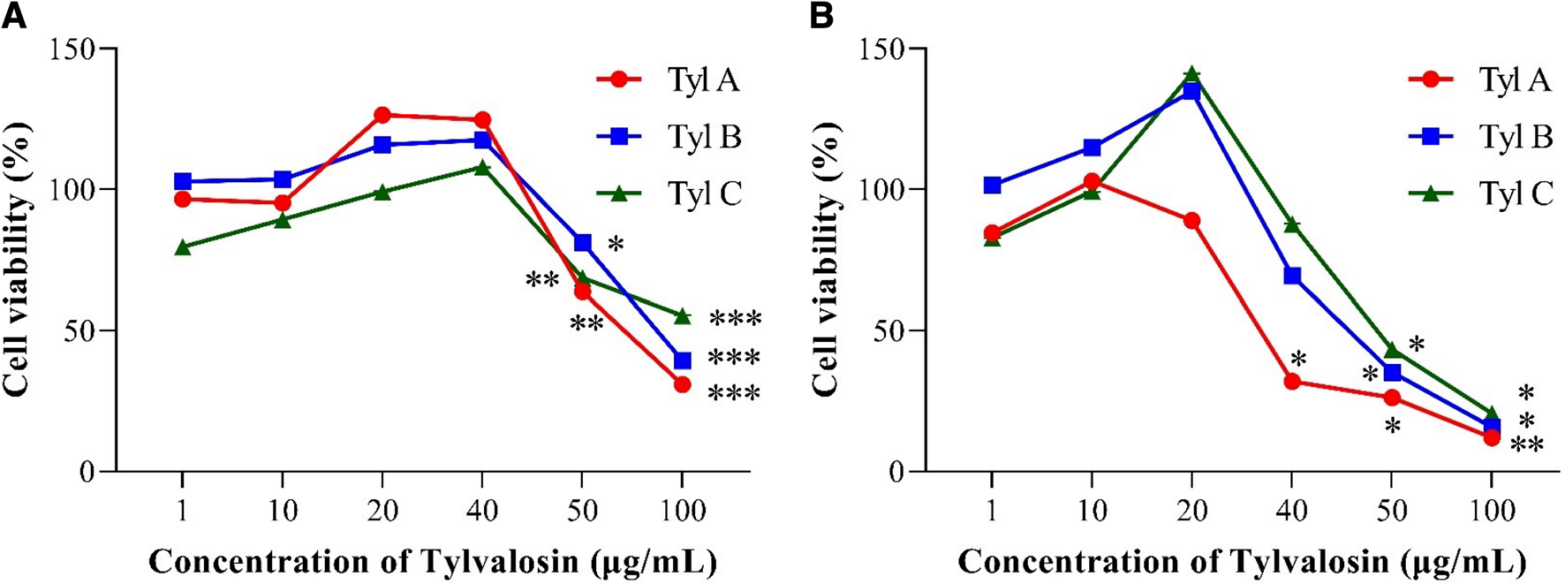
Cellular toxicity of three kinds of commercial Tylvalosin tartrates to MARC-145 cells and primary PAMs. Tylvalosin tartrates with different concentrations (1, 10, 20, 40, 50 and 100 µg/mL) in DMEM or RPMI-1640 with 2% FBS were added to both MARC-145 cells and primary PAMs for 36 h incubation. The cellular toxicity was determined by testing the cell viabilities of MARC-145 cells (**A**) and primary PAMs (**B**) via CCK-8, compared with the untreated cells. The data are shown as means ± SD (standard deviation), n = 3 independent experiments. Asterisks indicate a statistically significant difference, compared with untreated cells (*p < 0.05; **p < 0.01; ***p < 0.001)

To evaluate the cytotoxicity of Tylvalosin tartrate in vitro, the viability of MARC-145 cells and primary PAMs were tested after 36 h treatment with the Tylvalosin tartrate from three producers, by using the CCK-8 cell proliferation kit. The viability of MARC-145 cells is close to 100% till the concentrations of Tylvalosin tartrate reached 50 µg/mL (Fig. 2A). Similarly, the viability of PAMs significantly reduced, when the concentration of Tylvalosin tartrate reached 20 µg/mL for Tyl A or 40 µg/mL for both Tyl B and Tyl C (Fig. 2B).

### Tylvalosin tartrate inhibits PRRSV proliferation in vitro

**Fig. 3:**
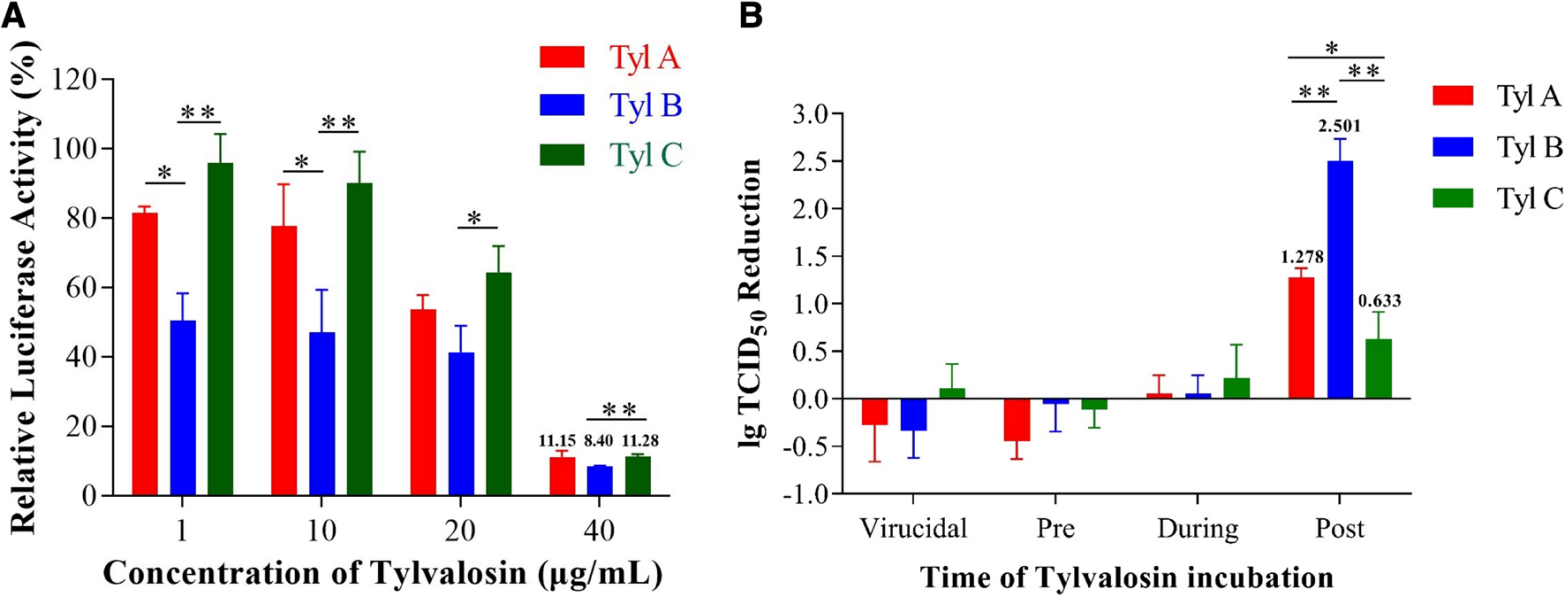
Tylvalosin tartrate inhibits PRRSV proliferation in vitro. **A** Relative luciferase activity of the NanoLuc-tagged PRRSV strain RvJX-Nsp2_325_-HiBiT in MARC-145 treated with Tylvalosin tartrate at different concentrations. **B** The viral titer reduction of PRRSV in MARC-145 cells with different Tylvalosin tartrates treating process. The data are shown as means ± SD (standard deviation), n = 3 independent experiments. Asterisks indicate statistically significant differences, between different kinds of Tylvalosin tartrate(*p < 0.05; **p < 0.01; ***p < 0.001)

To evaluate the inhibition effect of Tylvalosin tartrate on PRRSV replication, the relative luciferase activity of PRRSV RvJX-Nsp2_325_-HiBiT from cells treated with different Tylvalosin tartrate in a serial of concentration was tested. The data indicated these three products of Tylvalosin tartrates, especially Tyl A and Tyl B, can inhibit the proliferation of PRRSV at the concentration of 1 µg/mL. And the inhibition effect increased when the concentration reached 40 µg/mL, reducing the relative luciferase activity near to 10% of the untreated group (Fig. 3A).

Next, to investigate if the Tylvalosin tartrates can directly play a virucidal effect on the PRRSV or inhibit the early stage of PRRSV infection by a short-term treatment on the cells, previous to or during the viral inoculation, four different manners of Tylvalosin tartrates treatment were set and the relative reduction of viral titers were tested at 36 h post-inoculation. The reduction of logTCID_50_ compared with the mock group showed that Tylvalosin tartrates play the inhibition effect only via long-term action on the cells during the PRRSV proliferation and it could not achieve an antiviral effect only by 1-hour treatment before or during virus inoculation (Fig. 3B).

**Fig. 4:**
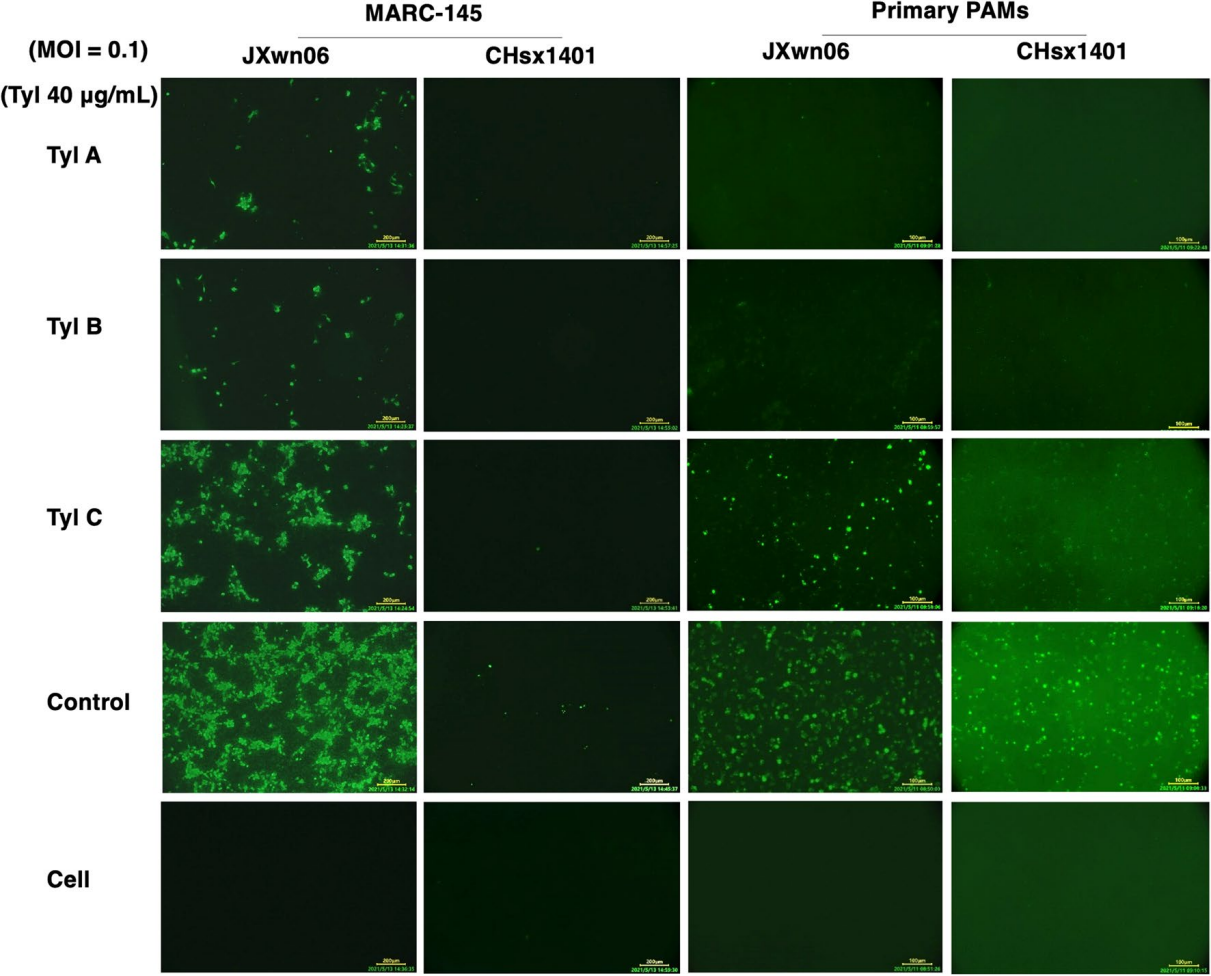
Detecting the Tylvalosin tartrates’ inhibition effect on PRRSV by IFA. MARC-145 cells and primary PAMs were respectively infected with JXwn06 or CHsx1401 and incubated with Tylvalosin tartrates at the concentration of 40 µg/mL respectively. At 36 h post-inoculation, the IFA was carried out to monitor the proliferation of PRRSV.

To further confirm the inhibition effect of Tylvalosin tartrate on different PRRSV strains, the representative HP-PRRSV strain JXwn06 and currently predominant NADC30-like field strain CHsx1401 were used to inoculate MARC-145 cells and PAMs, which were treated with Tyl A, Tyl B, and Tyl C at the concentration of 40 µg/mL respectively. At 36 h post-inoculation, the IFA was carried out to monitor the proliferation of PRRSV. As shown in Fig. 4, the three Tylvalosin tartrate products can inhibit the two PRRSV strains’ proliferation in both MARC-145 cells and PAMs, and the fluorescence signals of Tyl A and Tyl B treated groups were relatively less compared with that treated with Tyl C, as well the inhibition effect was higher on PAMs compared with that on MARC-145 cells.

### Differential transcription analysis of genes in MARC-145 cells treated with Tylvalosin and/or infected with PRRSV

**Fig. 5:**
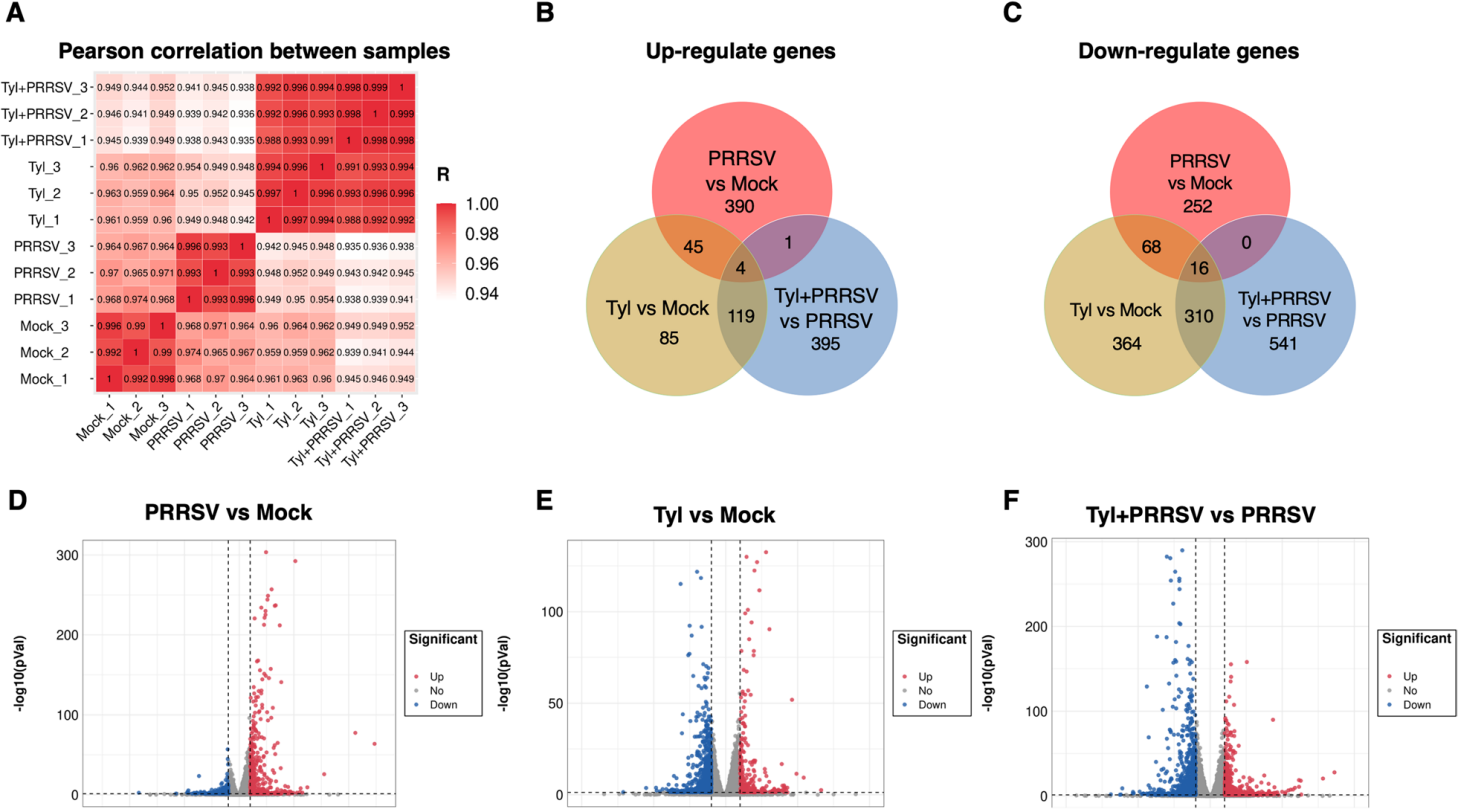
Differentially expressed genes in MARC-145 cells with PRRSV inoculation and/or Tylvalosin tartrate treatment. **A** Samples from three independently repeated experiments were submitted for RNA-seq, and the Pearson correlation between samples was displayed. The differentially expressed genes were selected with log2 (fold change) > 1 or log2 (fold change) < − 1 and with statistical significance (q value < 0.05) by the R package. Veen analysis was performed to identify the genes up-regulated or down-regulated in each group (**B** and **C**). And the volcano maps of distinguishable mRNA expression profiling in each group were shown (**D**,** E**, and **F**)

**Fig. 6:**
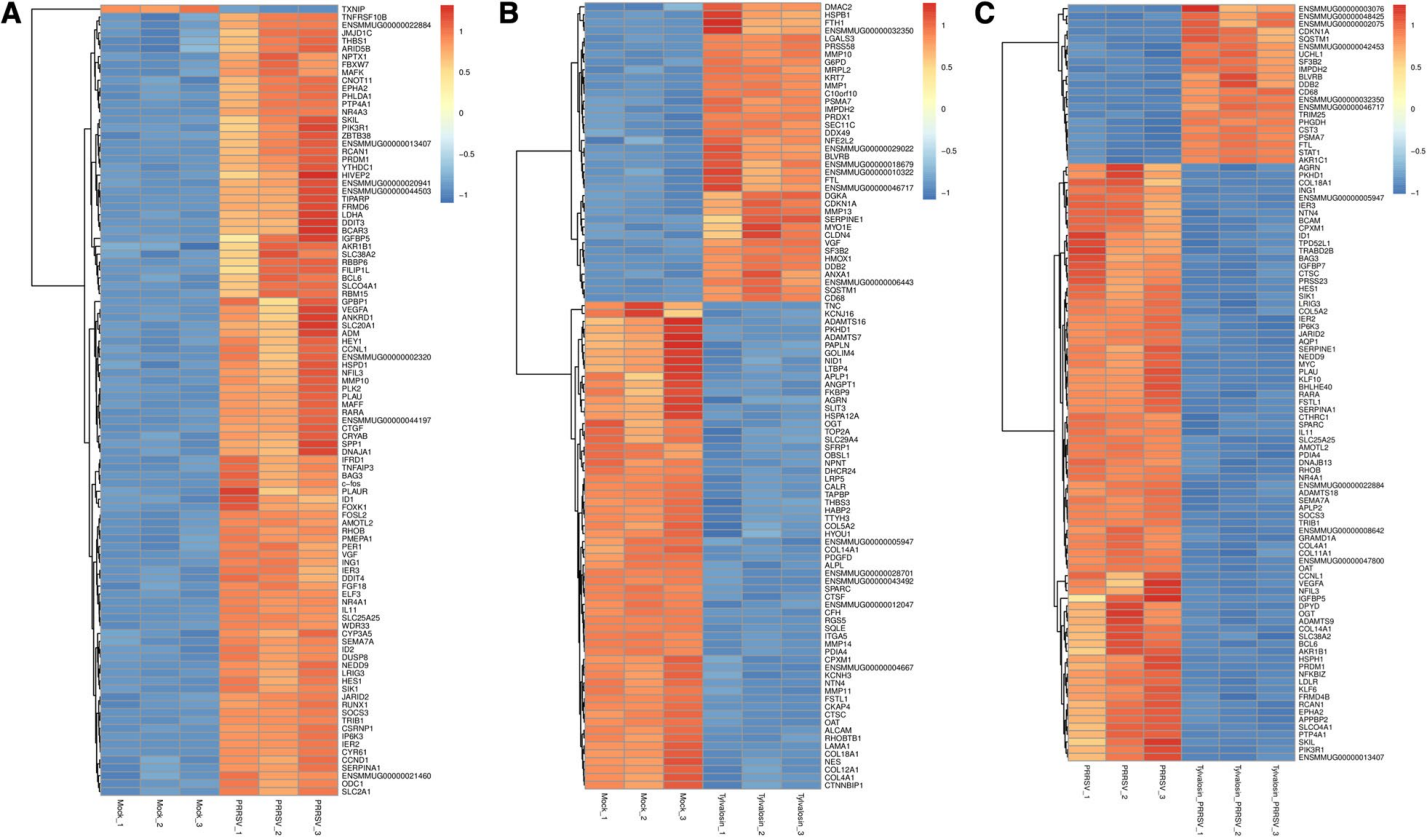
The heat map analysis of the top 100 differentially expressed genes (DEGs). The red color denotes the highly expressed up-regulated DEGs, and the blue color denotes down-regulated DEGs with lower expression levels. The gradation from red to blue represents the transition from large to small values of FPKM normalized log2 transformed counts

To initially find some clue to explore the anti-PRRSV mechanism of Tylvalosin tartrate, the RNA transcription profiles were obtained from Tylvalosin tartrate treated and/or PRRSV infected groups. The Pearson correlation coefficient analysis results indicate that the sequencing data among three intra-group repeat samples are well clustered (Fig. 5A). The transcriptome analysis results suggested that as many as 16,584 annotated genes were identified in all four groups, with 440 up-regulated and 336 down-regulated differentially expressed genes (DEGs) characterized between PRRSV infected group with Mock. Parallelly, the up-regulated/ down-regulated DEGs in the Tylvalosin tartrate treated groups were respectively 253/758 (Tylvalosin vs. Mock) and 519/867 (Tylvalosin + PRRSV vs. PRRSV), shown as VENN (Fig. 5B, C) and volcano maps (Fig. 5D–F). Furthermore, the top 100 differentially expressed genes (DEGs) with transcription level changes greater than 1.5-fold were shown in the heat map (Fig. 6).

### Analysis of Gene Ontology (GO) terms and Kyoto Encyclopedia of Genes and Genomes (KEGG) pathways of DEGs

**Fig. 7:**
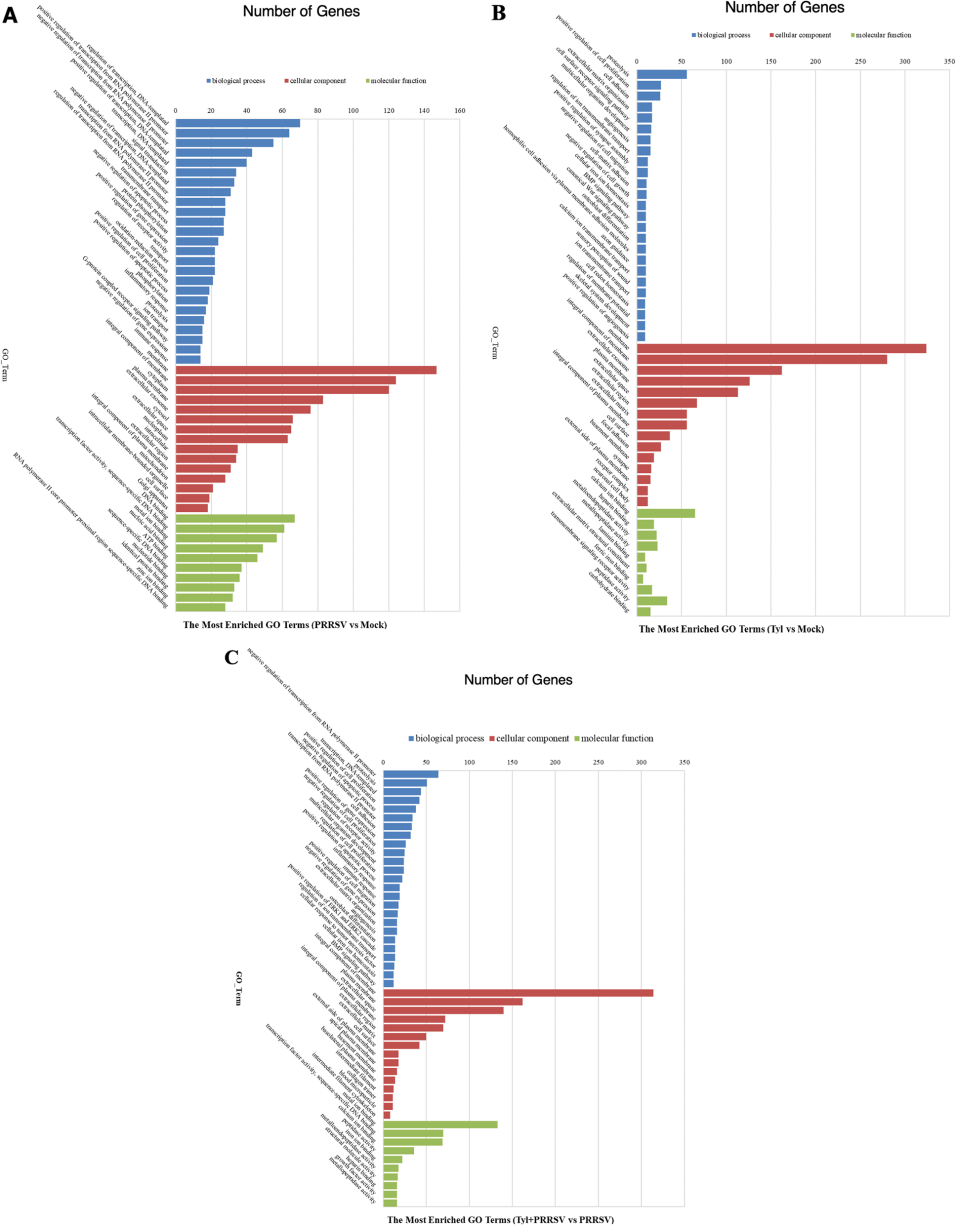
Gene Ontology (GO) terms enrichment of the differentially expressed genes (DEGs). The most significant enriched GO terms (top 50) among the DEGs in the groups of PRRSV versus Mock (**A**), Tylvalosin versus Mock (**B**), and Tylvalosin_PRRSV versus PRRSV (**C**) were shown

To further investigate the functions of DEGs related to Tylvalosin tartrate treatment and/ or PRRSV infection, the GO terms and KEGG pathways analysis were carried out, which might help explore the pathways related to the mechanism of Tylvalosin’s anti-PRRSV effect. The basic functions of the top altered DEGs were classified into three terms, including biological process, cellular component, and molecular function. When comparing the transcriptomic profiles between Tylvalosin tartrate treated cells and mock, the top biological processes of enriched GO terms include proteolysis, signal transduction, and oxidation-reduction process, and the major cellular components of DEGs can be mainly classified into the membrane, the integral component of the membrane and extracellular exosome, as well, the molecular function is mainly related with “binding”, such as to metal ion, calcium ion, and ATP. But, when comparing the PRRSV infected cells between Tylvalosin tartrate treated and untreated groups, it could be found that the number of DEGs in the biological process proteolysis, signal transduction, and the oxidation-reduction process is still arranged ahead, meanwhile, the genes involved in the regulation of transcription from both DNA and RNA are well enriched. Besides, the molecular related to the cellular component of the membrane and metal ion binding is still significantly enriched (Fig. 7A–C).

**Fig. 8:**
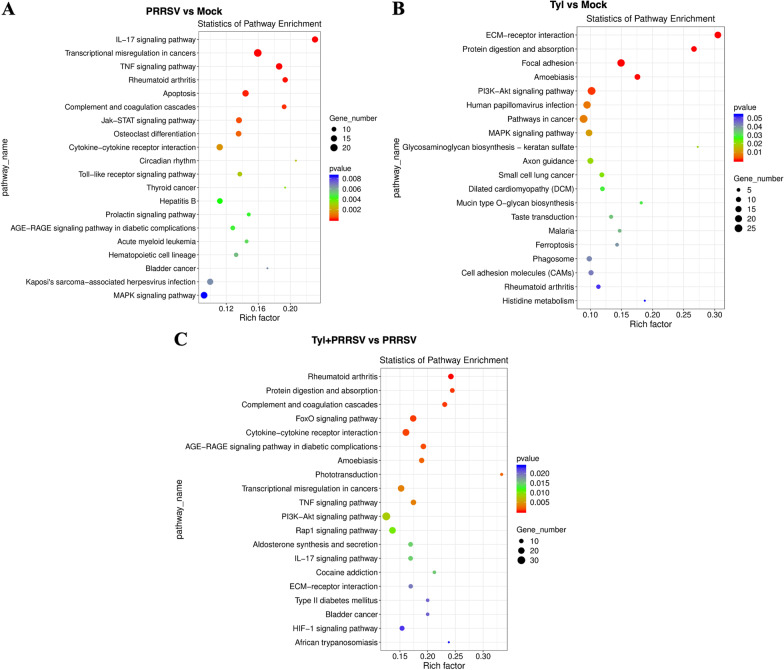
KEGG pathway enrichment of the differentially expressed genes (DEGs). The most significant enriched KEGG pathways among the DEGs in the groups of PRRSV versus Mock (**A**), Tylvalosin versus Mock (**B**), and Tylvalosin_PRRSV versus PRRSV (**C**) were shown

The altered pathways associated with Tylvalosin tartrate treatment were further analyzed by KEGG. When compared with Mock, the differential expressed genes in the Tylvalosin group were enriched to 262 signal pathways, including the ECM-receptor interaction, protein digestion, absorption, focal adhesion, and PI3K-Akt signaling pathway, which are well enriched. Meanwhile, rheumatoid arthritis (inflammation), protein digestion and absorption, complement and coagulation cascades, FoxO signaling pathway, and cytokine-cytokine receptor interaction were also well enriched, when comparing the PRRSV infected cells between Tylvalosin tartrate treated and untreated groups (Fig. 8A–C).

### Experimental validation of selected genes

**Fig. 9:**
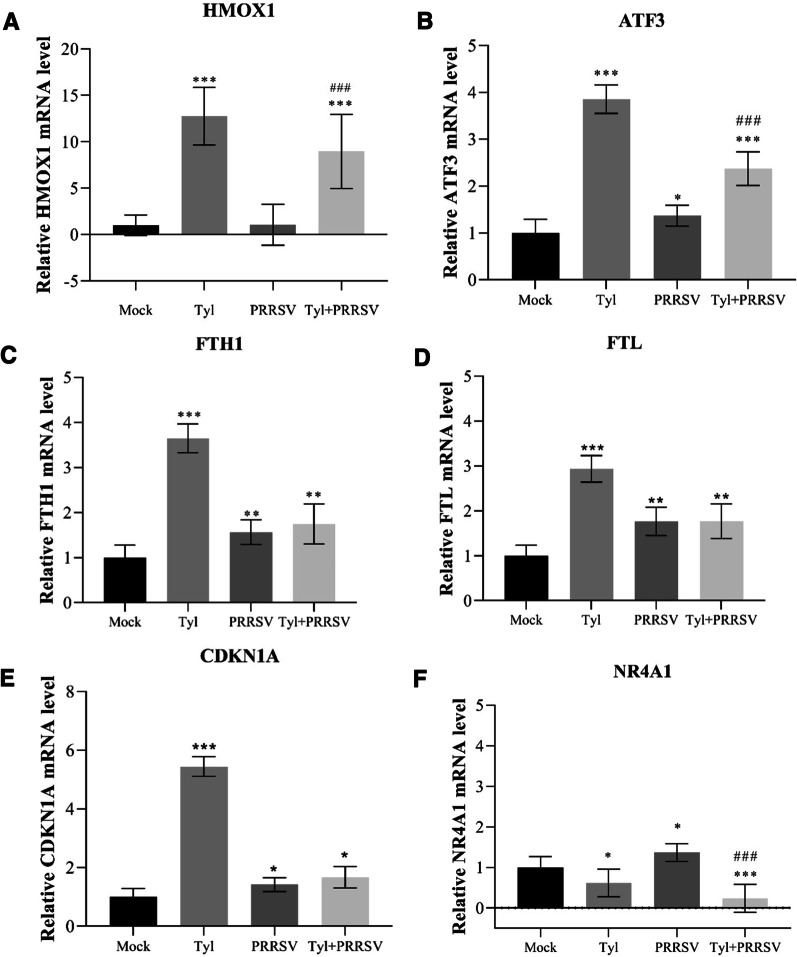
RT-qPCR validation of differentially expressed genes in MARC-145 cells with PRRSV inoculation and/or Tylvalosin tartrate treatment. Shown are the transcription levels of five up-regulated genes (**A**–**E**) and one down-regulated gene (**F**) in MARC-145 cells treated with Tylvalosin tartrate.The data are shown as means ± SD (standard deviation), n = 3 independent experiments. Asterisks indicate a statistically significant difference, compared with Mock group (*p < 0.05; **p < 0.01; ***p < 0.001), pounds indicate a statistically significant difference, compared with PRRSV inoculation group (###*p* < 0.001)

To validate the accuracy of transcription levels in RNA-seq, the mRNAs of six DEGs including HMOX1, ATF3, FTH1, FTL, NR4A1, and CDKN1A were selected to confirm the transcription levels in different treatment groups, by using RT-qPCR with the primers in Table 1. As shown in Fig. 9, the relative mRNA level of these genes is all regulated by the treatment of Tylvalosin tartrate, with a similar trend with the FPKM value in RNA-seq results. Among these molecular, the mRNA level of HMOX1, ATF3, FTH1 FTL, and CDKN1A in the Tylvalosin tartrate treated group is significantly higher than that in mock, but only the mRNA level of HMOX1 and ATF3 in Tylvalosin_PRRSV group are significantly higher than that in PRRSV infection group. Meanwhile, the NR4A1 in both Tylvalosin tartrate treated groups was significantly down-regulated.

**Fig. 10:**
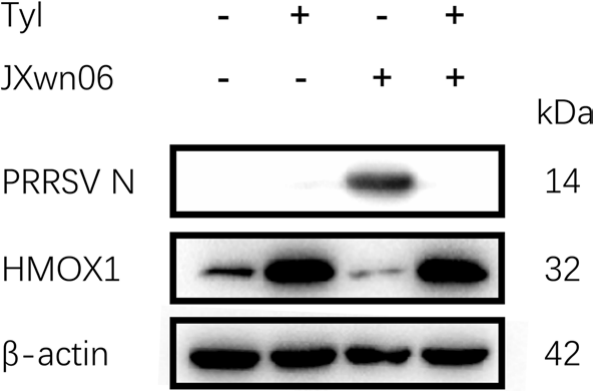
Western blot validation of PRRSV N protein and HMOX1 protein expression level in MARC-145 cells with PRRSV inoculation and/or Tylvalosin tartrate treatment at 24 h post-inoculation

HMOX1, which has been reported to show anti-PRRSV function in previous study, was selected to further identify the protein expression level by western blotting. The PRRSV was inhibit with the up-regulation of HMOX1 in Tylvalosin_PRRSV group (Fig. 10). This result shows similar trend with the results of PRRSV-inhibiting effect of Tylvalosin, and the transcription results in RNA-seq and RT-qPCR experiments above.

## Discussion

The porcine reproductive and respiratory syndrome is regarded as one of the most economically important diseases for the pork industry. The causative agent PRRSV is tagged for its genetic and antigenic variation, immunosuppression, persistent infection, increased secondary infection, and co-infection with other pathogens. Currently, the PRRSV MLV is regarded as a valuable tool for disease prevention and control in the field, however, as its unsatisfied heterologous cross-protection efficiency, the emergence of new strains will lead to outbreaks in PRRS stable or vaccinated herds. Besides, the PRRSV MLV can still replicate in the pig, causing viremia and virus shedding, raising the risk of reversion to virulence and recombination with field strains. Even though the killed vaccine is safer, the protection efficacy is still debatable. Therefore, it is necessary to develop new strategies to prevent and control this disease, especially to find some consistent methods, which can deal with all kinds of variated PRRSV strains.

Tylvalosin tartrate is a broad-spectrum anti-infective veterinary macrolide, which is currently widely used on pig farms to control respiratory and enteric bacterial pathogens, such as *Mycoplasma sp.* and *Lawsonia intracellularis *[18, 27]. As PRRSV is often complicated by the co-infected *Mycoplasma sp.*, causing serious pneumonia, Tylvalosin tartrates are usually used to attenuate PRRSV-induced clinical symptoms and improve the growth of pigs [20]. Indeed, Stuart et al. [22] surprisingly found that Tylvalosin tartrates also have a direct inhibiting effect on PRRSV, but the mechanism is still unclear.

In the present study, an in vitro inoculation model was set to evaluate the anti-PRRSV effect of Tylvalosin tartrates and determine the effectiveness of treatments with different drug concentrations or at different stages of viral infection. The results show that all three kinds of commercial Tylvalosin tartrates could inhibit PRRSV in a concentration-dependent manner, and their maximum safe concentration is around 40 µg/mL. To further identify the inhibiting stages, four different Tylvalosin tartrates treating processes were investigated and the results showed that Tylvalosin tartrates show no virucidal effect to “kill” the virus directly, and remaining the effective concentration of Tylvalosin tartrates in the cell medium for a period is essential for playing its anti-PRRSV role, as 1-hour-long incubation on the cells pre or during the virus infection cannot take effect. Generally, a lot of experimental factors can influence the evaluation results of antiviral potency, including viral strain, MOI of inoculation, cell line, treatment duration, the concentration of serum present in the cell culture medium, and methods for virus quantification, so the effective concentration might specifically vary among the different set of evaluation tests. Considering the environment in vivo is more complicated, many characteristics of drug and host factors can interrupt the anti-viral effect, such as drug absorption, distribution, and metabolism, animal experiments are further needed to explore whether it can effectively inhibit PRRSV in vivo and determine the effective concentration of Tylvalosin tartrates. Importantly, the results obtained in this study can provide valuable clues for translating the antiviral activity to the setting.

To further identify the intracellular processes and characterize the dynamic transcriptome landscapes that point to the mechanism associated with the anti-PRRSV activity of Tylvalosin tartrates, RNA sequencing and transcriptome analysis were carried out. Based on the GO terms and KEGG pathway analysis, we found that Tylvalosin tartrate can regulate the signal transduction, proteolysis, and oxidation-reduction process, as well as some pathways such as protein digestion and absorption, PI3K-Akt signaling pathway, FoxO signaling pathway and Ferroptosis pathways, which might relate to PRRSV proliferation or host innate immune response in the cells. These findings will provide valuable clues for further exploring the host cell restriction factors or anti-PRRSV target. More interestedly, several genes, which have been previously reported to associate with antivirus functions, are regulated by Tylvalosin tartrates. For example, the transcription of heme oxygenase 1 (HMOX1) is around 10 times up-regulated in Tyl or Tyl + PRRSV groups, and its encoding protein has been known as an inhibitor at the early stage of PRRSV infection as inhibiting virus-induced oxidative stress by activating the Nrf2-HMOX1 pathway [28, 29]. Our result proved that Tylvalosin tartrate could up-regulate the protein expression of HMOX1 with the inhibition of PRRSV proliferation, which suggests one possible antiviral mechanism of Tylvalosin tartrate. We also found another gene cyclin-dependent kinase inhibitor (CDKN1A), also known as P21, which can be degraded by PRRSV to promote the cells to enter into the S phase for facilitating the viral replication, and has been up-regulated [30]. Besides, several other genes that might be generally associated with antivirus functions, including exocrine component 5 (EXOSC5), which activates RNA binding activity and acts on the early or middle stage of viral defense response; signal transducer, and activator of transcription 1 (STAT1), an important signal molecular of IFNs pathway related with antiviral activity; NFKB inhibitor zeta (NFKBIZ), which can interact with NF-B protein through anchor protein repetitive domain, etc. [31–33]. The anti-PRRSV functions and the mechanism of these molecules during Tylvalosin tartrates treatment are worth to further study in the future.

## Conclusion

Under the maximum safe concentration (≤ 40 µg/mL), Tylvalosin tartrate can inhibit PRRSV proliferation in cells in a dose-dependent manner, and remaining at the effective concentration for a period is essential for playing its anti-PRRSV role. The transcriptomic data reveals the dynamic transcriptome profiles and functions of DEGs in Tylvalosin tartrate treated and/ or PRRSV infected cells, which provides important clues to understanding the mechanism of Tylvalosin tartrate’s anti-PRRSV effect.

## Data Availability

The PRRSV strains mentioned in this study are available in GenBank with the accession numbers: JXwn06 (EF641008) and CHsx1401 (KP861625).

## Abbreviations

PRRSV: Porcine reproductive and respiratory syndrome virus
PAMs: Pulmonary alveolar macrophages
PCV2: Porcine circovirus type 2
HP-PRRSV: Highly pathogenic PRRSV
RFLP: Restriction fragment length polymorphism
ORF5: Open reading frame 5
DMEM: Dulbecco’s modified Eagle’s medium
SPF: Specific-pathogen-free
MOI: Multiplicity of infection
IFA: Indirect immunofluorescence assay
McAb: Monoclonal antibody
FDR: False discovery rate
DEGs: Differentially expressed genes
GO: Gene Ontology
KEGG: Kyoto Encyclopedia of Genes and Genomes
HMOX1: Heme oxygenase 1
CDKN1A: Cyclin-dependent kinase inhibitor
EXOSC5: Exocrine component 5
STAT1: Signal transducer and activator of transcription 1
NFKBIZ: NFKB inhibitor zeta
